# *Strongyloides stercoralis* and other intestinal parasites in patients receiving immunosuppressive drugs in northern Iran: a closer look at risk factors

**DOI:** 10.4178/epih.e2021009

**Published:** 2021-01-20

**Authors:** Leila Mirzaei, Keyhan Ashrafi, Zahra Atrkar Roushan, Mohammad Reza Mahmoudi, Irandokht Shenavar Masooleh, Behnaz Rahmati, Farshid Saadat, Hamed Mirjalali, Meysam Sharifdini

**Affiliations:** 1 Department of Microbiology, School of Medicine, Guilan University of Medical Sciences, Rasht, Iran; 2 Department of Biostatistics, Faculty of Medicine, Guilan University of Medical Sciences, Rasht, Iran; 3 Rheumatology Research Center, Razi Hospital, School of Medicine, Guilan University of Medical Sciences, Rasht, Iran; 4 Foodborne and Waterborne Diseases Research Center, Research Institute for Gastroenterology and Liver Diseases, Shahid Beheshti University of Medical Sciences, Tehran, Iran

**Keywords:** Strongyloidiasis, Immunocompromised patients, Risk factors, Iran

## Abstract

**OBJECTIVES:**

The objective of this study was to evaluate the prevalence of *Strongyloides stercoralis* and other intestinal parasites in patients receiving immunosuppressive drugs in northern Iran and to investigate related risk factors.

**METHODS:**

This cross-sectional study was conducted among 494 patients receiving immunosuppressive drugs, including cancer patients undergoing chemotherapy (n=188) and those treated with prolonged corticosteroid administration (n=306). All fresh fecal samples were examined using the direct wet-mount, formalin ethyl acetate concentration, and agar plate culture techniques.

**RESULTS:**

In total, 16.8% of patients were positive for at least 1 intestinal parasite; the helminthic and protozoan infection rates were 5.1% and 12.3%, respectively. The infection rate was significantly higher in corticosteroid-treated individuals (19.6%) than cancer patients (12.2%) (p<0.05). The prevalence rate of *S. stercoralis* among patients receiving chemotherapy and those treated with corticosteroids were 4.3% and 5.2%, respectively. The prevalence rate of *S. stercoralis* infection was significantly higher in older patients (p<0.05).

**CONCLUSIONS:**

Strongyloidiasis is one of the most common parasites among patients receiving immunosuppressive drugs in northern Iran. Early diagnosis and proper treatment of these patients are necessary to minimize the complications of severe strongyloidiasis.

## INTRODUCTION

Intestinal parasitic infections (IPIs) are major causes of significant morbidity and mortality rates worldwide. Many of these infections occur in developing and poor countries due to insufficient access to health services, malnutrition, and poor sanitation [1]. Moreover, the number of people with immune deficiencies continues to increase yearly due to the spread of human immunodeficiency virus (HIV) and the prescriptions of immunosuppressive medications such as corticosteroids and chemotherapy drugs for autoimmune diseases [2]. These groups of patients are at a particularly high-risk for severely complicated infections with some parasitic agents.

*Strongyloides stercoralis* is a soil-transmitted helminth estimated to infect about 370 million people worldwide, especially in tropical and subtropical countries [3]. It is transmitted through the penetration of infective larvae into human skin when in contact with soil. Strongyloidiasis has variable manifestations, ranging from asymptomatic cases to serious clinical syndromes such as hyperinfection and disseminated syndrome. Most infected individuals are completely asymptomatic, while some have mild gastrointestinal, pulmonary, and cutaneous symptoms with or without fever [4,5]. Chronic strongyloidiasis may change to severe, complicated, and deadly strongyloidiasis in patients receiving corticosteroid and other immunosuppressive treatment, as well as those with diseases such as diabetes, hematologic malignancies, HIV infection, and human T-lymphotropic virus type 1 infection. Several parasitological methods have been used to detect *S. stercoralis* larvae in stool samples, and multiple studies have confirmed that nutrient agar plate culture is more sensitive than other parasitological techniques [6,7]. The incidence of severely complicated strongyloidiasis has dramatically increased over the 2 recent decades, mostly due to the growing number of immunocompromised patients [8]. Therefore, rapidly diagnosing chronic infections, updating epidemiological information, and screening people at risk are helpful measures to reduce the mortality and morbidity rate of strongyloidiasis [6,9].

*S. stercoralis* is endemic in the northern and southern coastal provinces of Iran due to the suitable moist environment, climatic, and geographic factors for the establishment of its life cycle [6,7,9,10]. Few studies have investigated infections of intestinal parasites, especially *S. stercoralis*, among immunosuppressed patients in Iran [11-13]. This study aimed to determine the frequency of *S. stercoralis* and other intestinal parasites among patients receiving immunosuppressive drugs in Guilan Province, in northern Iran, to provide a clear image of the current status of infections among these patients in this region.

## MATERIALS AND METHODS

### Study area and sample collection

**Figure 1.**
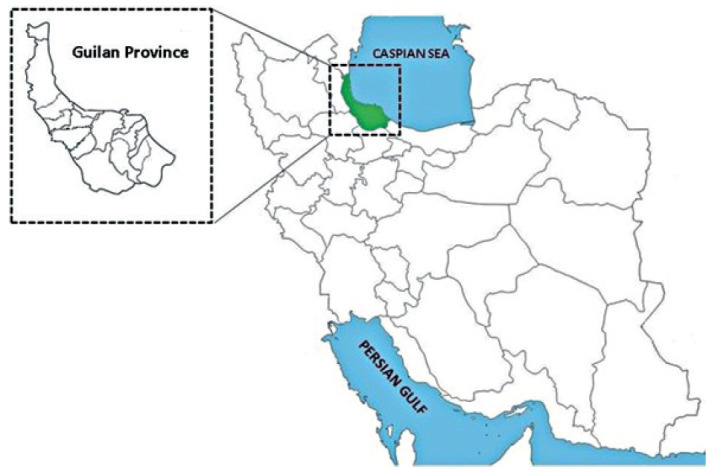
Map of Iran showing the geographical location of Guilan Province in northern Iran.

Guilan Province is located along the southern part of the Caspian Sea in the north of Iran (36° 34′–38° 27′ N, 48° 53′–50° 34′ E) (Figure 1). This region has a humid subtropical climate. The average annual precipitation, relative humidity, and annual temperature are about 1,506 mm, 80%, and 15.8°C, respectively. This province is geographically divided into coastal plains and mountainous forest regions [14,15]. The most common jobs of rural residents are rice and tea cultivation and animal husbandry, which can expose them to soil-transmitted and zoonotic helminths.

This cross-sectional study was conducted among 494 patients receiving immunosuppressive drugs, including cancer patients undergoing chemotherapy (n=188) and those treated with prolonged corticosteroid administration (n =306) from February 2018 to January 2019. Fresh stool samples were collected from referral hospitals in Guilan Province, including Razi, Rasool Akram, and Aria. Demographic data such as patients’ sex, age, job, and educational level were recorded through interviews.

### Stool examinations

All fresh fecal samples were examined using the direct wet-mount and formalin ethyl acetate concentration (FEC) techniques to detect the presence of any parasite. In addition, the agar plate culture (APC) technique was used to detect *S. stercoralis* infection, as described previously [7,9,10]. Briefly, about 3 g of a fresh stool sample was placed onto a nutrient agar plate and then incubated at room temperature for 2-3 days. Each plate was observed by stereomicroscopy to detect larvae and adult nematodes or their tracks. If the agar plate was positive, their surface was washed out by warm phosphate-buffered saline solution. The morphological characteristics of the parasites were then evaluated to identify and differentiate *S. stercoralis* from other possible nematodes such as *Trichostrongylus* spp., hookworms, and free-living nematodes [7,9,10].

The parasitic loads of *S. stercoralis*–infected patients were categorized into 3 groups: (1) low infection: FEC-negative with 1-4 larvae counted on the agar plate surface, (2) moderate infection: FEC-positive with 5-10 larvae counted on the agar plate surface, (3) high infection: FEC-positive with more than 10 larvae counted on the agar plate surface [7,9].

### Statistical analysis

Data processing and analysis were performed using SPSS version 18.0 (SPSS Inc., Chicago, IL, USA), employing the chi-square and Fisher exact tests. A p-value of less than 0.05 was considered to indicate statistical significance.

### Ethics statement

The protocol of this study was approved by the Ethics Committee of Guilan University of Medical Sciences, Iran (Ref. No. IR. GUMS.REC.1396.192).

## RESULTS

A total of 494 patients receiving immunosuppressive drugs were included, of whom 228 (46.2%) were male and 266 (53.8%) were female. In total, 83 (16.8%) patients were positive for at least 1 intestinal parasite, and the helminthic and protozoan infection rates were 5.1% and 12.3%, respectively. The prevalence rates of the parasites in cancer patients and corticosteroid-treated individuals were 12.2% and 19.6%, respectively. Statistical analysis revealed a significant difference between these 2 groups in terms of the presence of IPIs (p=0.03). The distribution of IPIs according to sex, age group, educational status, location, and occupation is illustrated in Table 1. No significant difference between these demographic factors and the presence of IPIs was found.

The most prevalent parasites were *Blastocystis hominis* (10.3%) and *S. stercoralis* (4.9%). The prevalence rate of these parasites is shown in Table 2. Mixed infections were observed in 6 individuals (1.2%).

The prevalence rates of *B. hominis* in cancer patients and corticosteroid-treated individuals were 6.4% and 12.7%, respectively, which constituted a significant difference (p=0.03).

**Figure 2.**
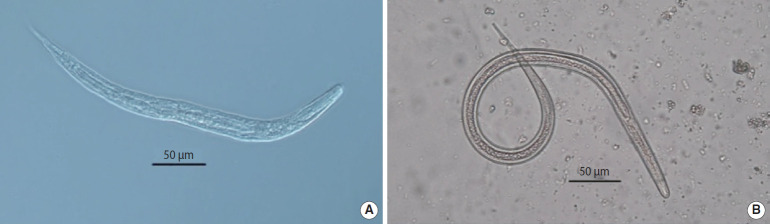
Rhabditiform (A) and filariform (B) larvae of *Strongyloides stercoralis* obtained through agar plate culture.

*S. stercoralis* was detected in 24 patients by at least one parasitological method (Figure 2). The prevalence rates of *S. stercoralis* among patients receiving chemotherapy and those treated with corticosteroids were 4.3% and 5.2%, respectively. No statistically significant difference was observed between the 2 groups regarding the prevalence of strongyloidiasis.

The prevalence of infection with *S. stercoralis* in the study population according to sex, age group, educational status, location, and occupation is illustrated in Table 3. A statistically significant difference in *S. stercoralis* infection according to age group (p<0.05). Other demographic characteristics failed to show any significant associations with the prevalence of *S. stercoralis* infection.

Among *S. stercoralis*-positive individuals, 7 (29.1%), 14 (58.3%), and 24 (100.0%) cases were detected by direct wet mount, FEC, and APC, respectively. Therefore, the APC method showed a 3.4 times and 1.7 times higher ability to detect the parasite than the direct wet-mount and FEC techniques, respectively.

In the positive cases, low, moderate, and high infection rates were detected in 10 (41.7%), 7 (29.2%), and 7 (29.2%) cases, respectively. No significant relationship was demonstrated between the parasitic load and sex, age group, and the 2 groups of immunosuppressed patients.

## DISCUSSION

Immunocompromised patients are at risk of serious and deadly infections with intestinal parasites in many countries, particularly in tropical and subtropical developing countries. In the current study, 16.8% of people taking immunosuppressive drugs in Guilan Province, northern Iran, were infected with intestinal parasites. Moreover, the overall prevalence of IPIs among corticosteroid-treated patients was significantly higher than that among cancer patients. It is now well established that both humoral and cellular immune functions are related to the psycho-neuroendocrine axis, in which any interaction via a prescribed corticosteroid disrupts immune responses against a parasite [16].

The prevalence rate of IPIs in cancer patients was 12.2%, which is higher than that previously reported for central Iran (6.7%) [17] and northwest Iran (10%) [18], yet significantly lower than that reported for Tehran, Iran (25.9%) [19], Yemen (63.1%) [20], southern Brazil (61.6%) [21], and Egypt (85.5%) among cancer therapy recipients with concurrent diarrhea [22].

Few studies have investigated the frequency of IPIs in patients receiving corticosteroid drugs worldwide. In this study, 19.6% of corticosteroid-treated individuals were positive for IPIs. This infection rate is lower than that previously reported in a study conducted in Egypt (92.3%) among children chronically treated with corticosteroids [23].

Our findings showed that *S. stercoralis*, with a prevalence rate of 4.9%, was a common intestinal parasite among the study population. Multiple epidemiological studies have illustrated that strongyloidiasis is common in the northern and southern regions of Iran [6,9,10,24], which is related to the temperate climate and the high humidity of these regions. The infection rate in endemic areas of Iran varies according to the target population and diagnostic methods employed. Most studies conducted in Iran were community-based and in immunocompetent populations [6,10,24,25]. The prevalence of *S. stercoralis* in the current study was similar to that found in a previous study carried out on rural inhabitants of Mazandaran Province using the APC technique (4.9%) [6]. Saeidinia et al. [24] reported a prevalence rate of 1.2% for *S. stercoralis* in institutionalized mentally disabled individuals in Guilan Province. Furthermore, a prevalence of 42% was reported for *S. stercoralis* among patients with eosinophilia in Guilan Province [26]. Another study among the residents of rehabilitation centers found an infection rate of 2.1% for *S. stercoralis* in Mazandaran Province [27]. Recently, the prevalence rate of this parasite was detected in 9.7% of residents of Khouzestan Province using nested polymerase chain reaction [10]. Despite the high prevalence of strongyloidiasis in endemic regions of Iran, there still remains no comprehensive understanding of its epidemiology and seroepidemiology in immunocompromised patients. Recently, Rafiei et al. [13] detected anti-*S. stercoralis* antibodies in 14.4% of immunocompromised patients in southwest Iran.

Several epidemiological studies in different groups of immunocompromised patients across the world have reported *S. stercoralis* using parasitological methods. The prevalence rate of this parasite in our study was higher than those reported previously for northeast India (3.2%) [28], Colombia (3.6%) [29], Brazil (2.4%) [30], and Saudi Arabia (2.2%) [31] when studied among various groups of immunocompromised patients. *S. stercoralis* was reported in 4.4% of cancer patients undergoing chemotherapy in southern Brazil [21]. Blatt et al. [32] reported that 10% of HIV-positive individuals were infected with *S. stercoralis* in Brazil.

In the current study, the prevalence rate of *S. stercoralis* among users of corticosteroid drugs was higher than that among cancer patients; however, the difference between the 2 groups was not statistically significant. Glucocorticoids, such as hydrocortisone, prednisolone, methylprednisolone, betamethasone, and dexamethasone, suppress eosinophilia and lymphocyte activation and reduce inflammation, thereby impairing the ability of the intestine to contain parasites [8,16]. Furthermore, some researchers have suggested that these drugs may directly affect parasites, accelerating the transformation from rhabditiform to invasive filariform larvae [8,16].

Our study showed that *S. stercoralis* infection was more prevalent in patients over 60 years of age than in other groups. This finding agrees with some previous studies that found a correlation between a higher prevalence rate of strongyloidiasis and increasing age [9,10,33]. This relationship may be attributed to the possibility of *S. stercoralis* autoinfections in infected individuals for several decades or even their entire lives. Furthermore, susceptibility to infection increases among older individuals due to the reduced efficiency of their immune response [34].

Our data showed that there was no significant relationship between *S. stercoralis* infection and sex. This finding is in agreement with some epidemiological studies [10,13,35]. However, several researchers reported that the rate of infection in males was higher than females due to a higher exposure of males to the source of infection as the result of working in rice and tea fields and gardening [9,33]. Other tested risk factors, including educational status, occupation, and location, were not associated with strongyloidiasis.

The application of a sensitive test for the diagnosis of strongyloidiasis is essential for high-risk individuals to decrease the mortality and morbidity associated with this infection. Several parasitological methods such as FEC, APC, Harada-Mori culture, and the Baermann method have been used to detect *S. stercoralis* larvae in stool samples [36]. Many studies have illustrated that APC is more sensitive than other parasitological methods for the diagnosis of *S. stercoralis* [6,7,36-38]. Our findings also confirmed that the sensitivity of APC was higher than that of other conventional methods. This culture protocol detected the parasite 3.4 times and 1.7 times more frequently than the direct wet-mount and FEC techniques.

In the present study, *B. hominis* was the most common infection in patients receiving immunosuppressive drugs (10.3%). This is similar to the findings of other studies that detected *B. hominis* as the most common parasite among cancer patients in Iran (22.3%) [19], immunosuppressed patients in Saudi Arabia (33.3%) [39], and immunosuppressed patients in Iran (4.2%) [17]. However, *B. hominis* is one of the most common parasites routinely found in human stool samples, and its pathogenicity is still highly controversial [40]. There are many potential sources for human infections, such as water resources, pets, and vegetables [41,42]. Its high prevalence is probably related to poor hygiene and the consumption of contaminated food and drinking water. In this study, the prevalence rate of this parasite in corticosteroid-treated patients was significantly higher than that in cancer patients.

Our results demonstrated a low-frequency rate of *Giardia lamblia* (0.8%). This protozoan is more common in children than in adults [43], and our study participants were over 40 years old; therefore, such a finding was expected. Epidemiological studies among cancer patients in Iran revealed that the frequency of *G. lamblia* was between 0.4% and 5.1% [18,44,45].

In our study, 1 case of infection with *Trichostrongylus* spp. was observed. According to recent studies, human trichostrongyliasis is prevalent in northern Iran due to close contacts between people and herbivorous animals [46-50].

Our study showed that *S. stercoralis* is one of the most common parasites among patients receiving immunosuppressive drugs in northern Iran. Early diagnosis and proper treatment of patients before chemotherapy or steroid therapy are necessary to minimize the complications of severe strongyloidiasis. Therefore, utilizing sensitive diagnostic methods such as APC for patients at risk, with careful attention to the elderly, in endemic areas will prevent the potentially fatal consequences of this nematode.

## Figures and Tables

**Table 1.** Prevalence of infections with intestinal parasites in patients receiving immunosuppressive drugs in Guilan Province, northern Iran, according to demographic factors

Variables	Positive, n (%)	Negative, n (%)	OR (95% CI)	p-value
Age (yr)				0.669
<40	12 (13.8)	75 (86.2)	1.00 (reference)	
40-60	43 (18.1)	195 (81.9)	0.80 (0.38, 1.67)	
>60	28 (16.6)	141 (83.2)	1.11 (0.65, 1.87)	
Sex				0.399
Female	41 (15.4)	225 (84.6)	1.00 (reference)	
Male	42 (18.4)	186 (81.6)	0.80 (0.50, 1.29)	
Educational status				0.672
Illiterate	23 (17.6)	108 (82.4)	1.00 (reference)	
Under diploma	43 (17.7)	200 (82.3)	1.29 (0.65, 2.55)	
Diploma and above	17 (14.1)	103 (85.8)	1.30 (0.70, 2.39)	
Occupation				0.676
Farmer	16 (19.8)	65 (80.2)	1.00 (reference)	
Government employee	2 (9.5)	19 (90.5)	1.02 (0.50, 2.11)	
Worker	9 (13.6)	57 (86.4)	0.44 (0.09, 2.03)	
Housewife	34 (16.0)	178 (84.0)	0.66 (0.28, 1.53)	
Other	22 (19.3)	92 (80.7)	0.79 (0.44, 1.44)	
Location				0.227
City	33 (14.4)	196 (85.6)	1.00 (reference)	
Village	50 (18.9)	215 (81.1)	0.72 (0.44, 1.17)	

OR, odds ratio; CI, confidence interval.

**Table 2.** Prevalence of infections with different species of intestinal parasites in patients receiving immunosuppressive drugs in Guilan Province, northern Iran

Intestinal parasites		n (%)
Single	*Giardia lamblia*	4 (0.8)
	*Entamoeba coli*	6 (1.2)
	*Blastocystis hominis*	46 (9.3)
	*Trichostrongylus* spp.	1 (0.2)
	*Strongyloides stercoralis*	20 (4.0)
Double	*Blastocystis hominis*+*Strongyloides stercoralis*	3 (0.6)
	*Blastocystis hominis*+*Giardia lamblia*	1 (0.2)
	*Blastocystis hominis*+*Entamoeba coli*	1 (0.2)
	*Trichostrongylus* spp.+*Strongyloides stercoralis*	1 (0.2)

**Table 3.** Prevalence of infections with *Strongyloides stercoralis* in patients receiving immunosuppressive drugs in Guilan Province, northern Iran, according to demographic factors

Variables	Positive, n (%)	Negative, n (%)	OR (95% CI)	p-value
Age (yr)				0.020
<40	1 (1.1)	86 (98.9)	0.12 (0.17, 0.99)	
40-60	9 (3.8)	229 (96.2)	0.43 (0.18, 1.03)	
>60	14 (8.3)	155 (91.7)	1.00 (reference)	
Sex				0.530
Female	11 (4.1)	255 (95.9)	1.00 (reference)	
Male	13 (5.7)	215 (94.3)	0.71 (0.31, 1.62)	
Educational status				0.088
Illiterate	11 (8.4)	120 (91.6)	1.00 (reference)	
Under diploma	9 (3.7)	234 (96.3)	2.65 (0.82, 8.58)	
Diploma and above	4 (3.3)	116 (96.7)	1.11 (0.33, 3.69)	
Occupation				0.080
Farmer	9 (11.1)	72 (88.9)	1.00 (reference)	
Government employee	1 (4.8)	20 (95.2)	2.72 (0.87, 8.46)	
Worker	1 (1.5)	65 (98.5)	1.09 (0.12, 9.83)	
Housewife	8 (3.8)	204 (96.2)	0.33 (0.03, 2.93)	
Other	5 (4.4)	109 (95.6)	0.85 (0.27, 2.67)	
Location				0.408
City	9 (3.9)	220 (96.1)	1.00 (reference)	
Village	15 (5.7)	250 (94.3)	0.68 (0.29, 1.58)	

OR, odds ratio; CI, confidence interval.
